# Relevance of VEGF and Nephrin Expression in Glomerular Diseases

**DOI:** 10.1155/2011/718609

**Published:** 2011-07-26

**Authors:** Claudia A. Bertuccio

**Affiliations:** Department of Cell Biology and Physiology, University of Pittsburgh, Pittsburgh, PA 15261, USA

## Abstract

The glomerular filtration barrier is affected in a large number of acquired and inherited diseases resulting in extensive leakage of plasma albumin and larger proteins, leading to nephrotic syndrome and end-stage renal disease. Unfortunately, the molecular mechanisms governing the development of the nephrotic syndrome remain poorly understood. Here, I give an overview of recent investigations that have focused on characterizing the interrelationships between the slit diaphragm components and podocytes-secreted VEGF, which have a significant role for maintaining the normal podocyte structure and the integrity of the filtering barrier.

## 1. Characteristic of the Glomerular Filtration Barrier and the Slit Diaphragm

 The cells of the human body are surrounded by liquid that is remarkably constant in its contents. The continued regulation of the dissolved compounds and ions in this internal environment is referred as *homeostasis*. Our kidneys are extremely important in preserving the homeostasis. They work rapidly and sensitively in order to regulate the excretion of unwanted and toxic products resulting from tissue metabolism, hence maintaining optimum concentration of the fluids, electrolytes, and acid-base in the body. The kidney is formed by an estimated number of renal glomeruli of ~1 million that filtrate approximately 180 liters of plasma every day, and only about 1.5–1.8 liters of this filtrate are excreted as urine. The plasma molecules are sieved through the renal glomerular filtration barrier based on their size and charge [1]. This barrier is a highly specialized structure which consists of three layers: a fenestrated endothelium, a 300–350 nm thick glomerular basement membrane (GBM), and the podocytes. The luminal side of the fenestrated endothelial cells is covered by a layer of negatively charged membrane-bound macromolecules (proteoglycans, glycosaminoglycans, glycoproteins, and glycolipids) named *glycocalyx*, and it is important for the selectivity properties of the glomerulus [2]. The GBM is composed of glycoproteins including type IV collagen, laminins, entactin/nidogen, and heparin sulphate proteoglycans such as agrin and perlecan. Type IV collagen and laminins form complexes which are connected by entactin/nidogen. This association plays an essential role in size selectively restricting the passage of the proteins through the glomerular filter [3–5]. The podocytes are highly differentiated cells, and they are the visceral epithelial cells of the kidney glomerulus. The podocytes project primary processes from the cell body, which further branch into numerous finger like processes called foot processes that enwrap the glomerular capillaries. The neighboring foot processes derived from different cell bodies are connected by a continuous adherent junction structure called the *slit diaphragm* (SD) [6]. The apical membrane above the SD is highly negatively charged mainly due to a heavily sialylated glycoprotein podocalyxin [7]. The basal membrane of podocyte foot processes serves to anchor podocytes to the GBM via several adhesion molecules including *α*3*β*1-integrin complex [8] and *α*- and *β*-dystroglycans [9]. On the other hand, the structure of the podocyte is maintained by a complex cytoskeleton consisting of actin, myosin, *α*-actinin-4, talin, and vinculin [10]. When podocytes are injured, podocytes undergo a dramatic morphological change named *foot process effacement *resulting from alteration in its cytoskeletal structure and intercellular junctions. A direct correlation between this process and the development of proteinuria both in human disease and in experimental models has been shown [11]. 

 The slit diaphragm expresses several members of the immunoglobulin (Ig) and cadherin superfamilies. Nephrin, a member of the Ig superfamily, is a transmembrane protein of 180 kDa in humans, and it is posttranslationally modified by N-glycosylation [12]. It has an extracellular segment containing 8 Ig-like domains, a fibronectin domain and the 10 putative N-glycosylation sites, a transmembrane region, and a short cytoplasmic segment [12–14]. It has been shown that the N-glycosylation of nephrin is important for its appropriate folding and localization in the plasma membrane [15]. In 1998, it was suggested that nephrin is the structural backbone of the slit diaphragm [12]. Several studies have provided evidence that nephrin associates with itself [16]. Nephrin has three “free” cysteines in the extracellular domain, one in the Ig motif 1, one in the spacer region between Ig motifs 6 and 7, and one in the fibronectin domain. These free cysteines residues in the extracellular domain allow the alignment between nephrin molecules from the neighboring foot processes in an antiparallel mode and bridge the distance between interdigitating podocyte foot processes [14, 17]. The cytoplasmic region of nephrin contains nine conserved tyrosines residues, which could become phosphorylated during the binding of nephrin ligand and can serve as binding sites for the SH2 domains of cytoplasmic targets [18–21]. The natural extracellular ligands for nephrin remain unknown until now. In addition to the glomerular podocytes of kidneys, nephrin is expressed in the pancreas and in the nervous system [22]. Humans and rodents also express a splice variant of nephrin that excludes the transmembrane region [23]. Mutations of the nephrin gene,* NPHS1, *produce *congenital nephrotic syndrome of the Finnish type* (CNF) [12]. CNF is an autosomal, recessive disorder, characterized by massive proteinuria in utero, and the classical signs of nephrotic syndrome hypoalbuminemia, hyperlipidemia, and edema developed within days after birth. Kidney biopsy of children with CNF shows podocyte foot process effacement and absence of the slit diaphragms [24]. The disease usually leads to death in the neonatal period but can be treated with dialysis followed by kidney transplantation in early childhood [25].

 A growing number of proteins, including Neph1, the classical P-cadherin that belongs to the Ig superfamily [6], FAT-1 a nonclassical cadherin superfamily member [26], and the tight junction protein ZO-1 [27] are localized at the SD. In the SD, the cytoplasmic tail of nephrin interacts with the adapter protein CD2-AP [28, 29], podocin [30, 31], and the actin cytoskeleton via CD2-AP [32]. The C-terminal domain of podocin interacts with nephrin, Neph1 [33], and CD2-AP [30]. Neph1 also interacts with podocin [33] and ZO-1 [34]. The canonical transient receptor potential 6 ion channel (TRPC6) interacts with podocin and nephrin in the slit diaphragm [35]. Subsequently, it was shown that mutations in several of these slit diaphragm-associated proteins, including Neph1 [36], podocin [37], CD2-AP [29], FAT-1 [38], and TRPC6 [39] results in effacement of podocytes, heavy proteinuria, development of nephrotic syndrome, and early postnatal death in the majority on the cases. In addition, mutations in *α*-actinin-4 [40], *α*3*β*1-integrin [8], and podocalyxin [41] can lead to the same phenotype. Taking together all these studies, it has been proposed that the slit diaphragm plays a major role in maintaining the filtration barrier of the renal glomerulus. However, the molecular components and function of the SD remain incompletely characterized.

## 2. Altered VEGF-A and Slit Diaphragm Protein Levels Can Cause Proteinuria

 Several lines of evidence showed that vascular endothelial growth factor A (VEGF-A), the best characterized angiogenic/vasculogenic factor, is a critical “cross-talk” protein among the three components of the glomerular filtration barrier [42]. Patients who underwent chemotherapy with anti-VEGF (e.g., bevacizumab, sunitinib) or receptor tyrosine kinase inhibitors lead to proteinuria, podocyturia, hypertension, edema, glomerular capillary endotheliosis, and glomerular thrombotic microangiopathy indicating damage to the glomerular filtration barrier [43–46]. In addition, several reports showed that women with preeclampsia, a condition characterized by excess circulating levels of a soluble form of the VEGF receptor (sFlt-1, see below), developed similar symptoms that are in VEGF ablation therapy [47, 48]. Experiments with normal healthy mice showed that intravenous infusion of sFlt-1 or anti-VEGF antibodies causes glomerular endotheliosis and proteinuria [49]. Taken together, these results emphasize the importance of the regulation of VEGF-A signaling in the development of proteinuria. 

 VEGF-A belongs to a family of multipotent cytokines. Six different VEGF-A isoforms and two known families can be generated by alternative splicing of eight exons separated by 7 introns of a single gene. The VEGFxxx family of isoforms, where xxx refers to the number of amino acids, is formed by differential splicing in exons 6 and 7 and the proximal splice site in exon 8 (termed exon 8a). They are named VEGF 121, 145, 165, 183, 189, and 206 based on the number of amino acids in humans. Rodents present the same number of isoforms, although they are one amino acid shorter [50]. VEGF_121_, VEGF_165_, and VEGF_189_ are the most abundantly expressed isoforms, whereas VEGF_145_ and VEGF_206_ are comparatively rare [50]. In human kidneys, the most abundant isoforms of VEGF-A are strongly expressed in podocytes and also in proximal and distal tubular cells [51]. Receptors for VEGF-A are vascular endothelial growth factor receptor VEGFR-1 (also known as Flt-1) and VEGFR-2 (also known as Flk-1), which are predominantly expressed on the glomerular endothelial cells and pre- and postglomerular vessels [51, 52]. These VEGF receptors belong to the PDGF (platelet-derived growth factor) family of receptor tyrosine kinase (RTKs, class III) and have a similar domain structure [53]. VEGFR-1 and -2 contain three functional domains: seven Ig-like extracellular domains, a single membrane spanning region, and a cytoplasmic tyrosine kinase domain [54, 55]. Binding of growth factors to the second and third immunoglobulin domains leads the receptors dimerization, protein kinase activation, autophosphorylation, and initiation of signaling pathways [56]. In addition to these receptors tyrosine kinases, VEGFxxx interacts with a family of coreceptors, the neuropilins (Np) [57]. Soluble truncated forms of both VEGFR-1 and VEGFR-2 have recently been found in plasma. Even though little is known about the latter [58], the soluble form of the VEGF receptor-1 (sFlt-1) is known to regulate VEGF activity by binding VEGF-A in the circulation [59]. Park et al. proposed that full length VEGFR-1 and sFlt-1 have a “decoy” function on the vascular endothelium, where they exert a negative effect on VEGF activity, by preventing VEGF binding to VEGFR-2 [60]. 

 Recently, another novel set of VEGF-A isoforms called “VEGFxxxb” have been described. The VEGFxxxb family is formed by splice variant using a distal site on exon 8 (termed exon 8b) [61, 62]. The VEGFxxxb transcripts code for polypeptides with the same length as the classical ones, because exon 8a and exon 8b have the same size. Therefore, these isoforms were named VEGF121b, 145b, 165b, 183b, 189b, and 206b. Furthermore, it has been identified that VEGFxxxb interacts with the VEGFR-2 extracellular domain, resulting in differential downstream kinase activation, but the “b-isoforms” are unable to bind Np [63]. VEGF165b was the first member of the VEGFxxxb family to be identified and was discovered in human renal cortex [62]. This isoform is expressed early in human kidney embryonic development and is expressed endogenously in differentiated, but not dedifferentiated human podocytes [64]. Schumacher et al. demonstrated that in normal renal development, the capillary loop stage showed the highest VEGF165 expression that diminishes afterward with a switch in favor of the VEGF165b isoform, which showed its highest expression in adult control glomeruli [65]. Several reports have shown that the inclusion of exon 8a results in the conventional proangiogenic and propermeability of VEGFxxx family but replacement of 8a with exon 8b in the VEGFxxxb family produces peptides that are antiangiogenic, inhibits permeability chronically, and reduces rather than promotes tumor growth [66–68]. In renal-cell [62], prostate [69], colon carcinoma [70], and malignant melanoma [71], VEGF165b has been reported to be downregulated. A dysregulation of VEGFxxxb expression was also observed in the glomeruli of humans with Denys-Drash syndrome [65] and in human preeclamptic placentas [72].

 In addition to the renal lesion reported in preeclampsia, altered levels of VEGF-A protein and/or mRNA were detected in various human and rodent experimental renal disease models associated with proteinuria. Diabetic nephropathy is characterized by the development of proteinuria followed by decreased glomerular filtration in association with glomerulosclerosis. Researchers have observed that urinary VEGF [73, 74], renal and/or glomerular VEGF levels and VEGF receptors are consistently increased in diverse experimental models of diabetes, especially early in the course of the disorder [75, 76]. *In vitro*, exposure of cultured podocytes to high glucose concentrations also upregulates VEGF expression [77]. Blocking the increased VEGF-A with anti-pan-VEGF antibodies improves the diabetes-related early renal dysfunction [73, 78]. Consequently, the inhibition of upregulated VEGF and its receptors resulted in beneficial effects on diabetes-induced functional and structural alterations.

 Several types of glomerulonephritis, in which experimental models or human kidney biopsies were analyzed, showed contradictory results. Glomerulonephritis (GN) are inflammatory glomerular diseases associated to proteinuria, frequently leading to chronic disease progression and renal replacement therapy. In some experimental models of human minimal change nephropathy (MCN), VEGF and its receptors were, as in diabetic nephropathy, upregulated and correlated with the severity of proteinuria [79]. In patients with MCN and nephrotic syndrome, the urinary VEGF levels are increased and positively correlated with the degree of proteinuria [80]. *In situ* hybridization studies revealed that VEGF mRNA expression was upregulated in podocytes with MCN [81]. Patients with congenital nephrotic syndrome of the Finnish type showed a slightly increased VEGF expression in glomeruli [82]. Recently, Hohenstein et al. have found increased glomerular VEGF expression in biopsies from patients with endocapillary nephritis, membranoproliferative glomerulonephritis (MPGN), and crescentic nephritis [83]. Biopsies from patients with membranous glomerulonephritis (MGN) presented markedly increased VEGF protein in podocytes [83]. Depending on the degree of the injury and inflammation in the glomeruli with glomerulosclerosis, VEGFR-1 expression was increased on various cell types in focal areas, and VEGFR-2 expression was more prominent in podocytes of biopsies with GN [83]. In contrast, other studies found either a reduction or lack of changes in the VEGF-VEGFR system in experimental or clinical studies of MGN [81, 84], MPGN [85, 86], crescentic glomerulonephritis [87], or focal segmental glomerulosclerosis (FSGS) [76]. Müller-Deile et al. reported a clinical case with metastatic cancers, where after total tumour nephrectomy, the patient developed FSGS and podocyturia in association with tyrosine kinase inhibitor treatments [88]. The downregulation of VEGF expression was also reported in adriamycin nephropathy rats [89] and puromycin aminonucleoside rats [90] with the progressive aggravation of foot processes morphology and development of proteinuria. These results suggest that VEGF might exert multiple effects on the glomerular pathophysiologic processes and that the expression balance of VEGF is essential for preserving the normal glomerular filtration function. 

 To demonstrate that VEGF-A signaling is a key for proper maintenance of the glomerular filtration barrier, several animal studies have been performed. Eremina et al. generated mice with gain or loss of function of VEGF specifically in the podocyte [91], avoiding the embryo-lethal effects observed in VEGF-A knockout [92] and in haploinsufficient mice [93]. Mice with podocyte-specific deletion of both VEGF alleles died at birth or within 18 hours of birth with kidney failure and grossly abnormal glomeruli. The glomeruli of these mice fail to form functional filtration barriers due to major defects in endothelial cell migration, survival, and differentiation. Mice with podocyte-specific heterozygosity for VEGF developed proteinuria, endotheliosis, and bloodless glomeruli, characteristic findings in patients with preeclampsia, which progressed to nephrotic syndrome and followed by kidney failure [91]. Kitamoto and colleagues administered VEGF-blocking antibodies to murine pups and observed that the development of the kidney was impaired in terms of glomerulogenesis and nephrogenesis [94]. Conversely, the overexpression of VEGF-A in podocytes also resulted in a dramatic phenotype due to a collapsing glomerulopathy and end-stage renal failure [91]. All these discoveries emphasize the role of VEGF-A in renal glomeruli and demonstrate that a tight regulation of VEGF-A signaling is required for development and maintenance of the glomerular filtration barrier.

 Different groups have examined the expression of slit diaphragm proteins in acquired glomerular diseases where the VEGF levels are altered. In both experimental and human diabetes, one of the several diseases involving a podocyte injury, various groups have found nephrin in the urine, downregulation of nephrin expression in the slit diaphragm and decreased nephrin mRNA levels that inversely correlated with the degree of proteinuria [95–98]. Moreover, no changes in the expression of CD2AP and podocin in podocytes from diabetic patients were observed [97]. However, reduction of the elevated concentrations of glycated albumin in diabetes animals showed significant reduction of proteinuria and restoration of distorted glomerular nephrin and VEGF expression [99]. Chun-Liang et al. demonstrated that activation of Notch-1 signaling in high glucose (HG)-treated human podocytes and in the glomeruli of diabetic rats produces increased VEGF expression, nephrin downregulation, and increased apoptosis, while the inhibition of Notch-1 signaling significantly abrogated VEGF activation, nephrin repression, and ameliorated proteinuria in HG-stressed cells and in diabetic kidney [100]. Garovic et al. reported that proteinuria in patients with preeclampsia is associated with downregulation of podocyte foot process proteins, as nephrin and synaptopodin [101]. Koop et al. and Fukuda et al. reported reduced nephrin expression at protein and/or mRNA level in patients and animals with MCN, and a significant decrease in the expression of podocin, podocalyxin and CD2AP was also seen in MCN [102–104]. Nevertheless, the genetic analyses of patients with MCN revealed heterozygous amino acid changes in nephrin and podocin, but no amino acid substitutions were detected in Neph1 and CD2AP genes [105, 106]. On the other hand, several reports have investigated the expression of nephrin in others glomerulosclerosis diseases, which were not always consistent. Furness et al. [107], Huh et al. [108], Srivastava et al. [109], Wang et al. [110], and Doublier et al. [111] reported a downregulation in nephrin expression in patients with focal segmental glomerular sclerosis, MPGN and membranous nephropathy (MN), whereas, as reported by Patrakka et al. [112], Guan et al. [113], and Hingorani et al. [114], no significant changes were seen in expression of nephrin at all. Hara et al. showed that blockage of VEGF activity in rats with progressive GN resulted in massive urinary protein excretion and downregulated expression of nephrin [115]. No extensive changes in the expression of others slit diaphragm proteins were observed in glomerulosclerosis [116, 117]. These discrepant results in some nephrotic syndromes may be attributed to differences in the degree of glomerulosclerosis and the methods used for detecting protein expression. Clearly, these studies demonstrate that disruption in VEGF levels are not only associated with increased proteinuria but also with readjustment of nephrin-dependent slit diaphragm structure. VEGF-nephrin signaling in podocytes may act through one or more survival signaling pathways, but these pathways are not full known.

## 3. Paracrine or Autocrine Podocytic VEGF-VEGFR System?

 During fetal development, the podocytes are one of the cell types producing the largest amounts of VEGF-A. When podocytes are fully differentiated, they continue to express VEGF-A, even though absolute levels of expression decrease [118]. The adjacent and nearby associated glomerular endothelial cells express VEGFR-1 and VEGFR-2 tyrosine kinase receptors [42, 91]. As a consequence, some authors have speculated that in normal conditions, a paracrine regulatory mechanism where the podocyte-secreted VEGF can bind to VEGF receptors on mesangium or glomerular endothelial cells. It is now broadly accepted that VEGF must move in the opposite direction of the glomerular filtrate in order to bind to its receptors. This notion is supported by the following studies. Quaggin group postulated that since podocytes produce the constitutive highest levels of VEGF, there is a concentration gradient favoring diffusion of VEGF from the podocyte to the glomerular endothelial cells in addition to the finding that the diffusion rate across the glomerular basement membrane is the predominant transport mechanism for solutes with the molecular radii under 30 Å while that of VEGF is about 26 Å [45, 119]. 

 Sison et al. [120] have found that induced whole-body postnatal deletion of VEGFR-2 caused marked damage in glomerular microvascular beds with loss of viable endothelial cells, whereas the same authors have observed nonthrombotic microangiopathy effect in the glomeruli of mice with whole body postnatal deletion of VEGFR-1 [120]. Eremina et al. showed that genetic inhibition of VEGF in mature podocytes also resulted in glomerular endothelial cell damage and thrombotic microangiopathy [45]. In addition, Sison et al. observed that the deletion of VEGFR-2 specifically from podocytes never developed proteinuria or glomerular injury, and the glomerular filtration barrier was intact [120]. These observations support the idea that VEGF signaling through VEGFR-2, not through VEGFR-1, is essential for maintaining the glomerular filtration barrier via a paracrine mechanism. 

 Nephrin cytoplasmic domain has a series of conserved tyrosine-based motifs [18], which are phosphorylated by Fyn kinase and are able to bind and phosphorylate the Nck adaptor [19] and the cytoplasmic kinase phosphatidylinositol-3-OH kinase (PI3K) proteins via their SH2 domains [20]. Phosphorylation of Nck contributes to the reorganization of the actin cytoskeleton in podocytes [21], and phosphorylated PI3K stimulates the serine-threonine kinase AKT signaling, leading the phosphorylation of molecules involve in the prevention of apoptosis and safeguard viability in podocytes [20]. Interestingly, Foster et al. postulated that VEGF treatment caused reduction of apoptosis through nephrin phosphorylation together with a decrease in AKT-signaling [121]. Sugimoto et al. observed that a single intravenous infusion of anti-VEGF antibodies into normal healthy mice produced excessive albumin excretion in the urine, massive glomerular, endothelial cell damage, and significantly reduction of nephrin expression [49]. Also, Foster et al. provided the first evidence that in podocytes, VEGF is associated with the cell membrane in normal human glomeruli [121]. This phenomenon could be explained either by an accumulation of VEGF protein before secretion or by the sequestration of VEGF, via receptor binding, onto the podocyte cell surface [122]. To answer these issues, the same group observed that the addition of type III receptor tyrosine-kinase inhibitor abolished the reducing cell death induced by VEGF, suggesting that a tyrosine kinase-mediated receptor for VEGF-A exists on podocytes [122]. These results suggest that podocytes may have the potential to bind the VEGF that they secrete. This hypothesis is also supported by other independent groups. They observed that, using transmission electron microscopy, VEGFR-2 is expressed in mice podocytes *in vivo* [123, 124]. Guan et al. observed that VEGFR-2 mRNA levels are increased in mouse-differentiated cultured podocytes [125], and recombinant VEGF_165_ induced VEGFR-2 mRNA and protein levels and reduced apoptosis in these differentiated podocytes. In contrast, VEGFR-2 was not detected in conditionally immortalized human podocytes [122]. These discrepancies may be species related. Nonetheless, Müller-Deile et al. identified that VEGFR-2 is the main receptor responsible for the PI3K/AKT regulation and podocyte survival in response to autocrine levels of VEGF-A and VEGF-C in human podocytes *in vitro* [126]. VEGF-A signaling also showed the ability to regulate slit diaphragm proteins by inducing podocin upregulation and increasing its interaction with CD2AP *in vitro* [125]. We have recently demonstrated that transgenic mice overexpressing VEGF_164_ in podocyte showed proteinuria, glomerular basement membrane thickening, loss of slit diaphragms, podocyte effacement, induced VEGFR-2 phosphorylation, and downregulation of nephrin expression [123]. Moreover, we provided the first evidence that nephrin, a podocyte-specific protein, and VEGFR-2 are associated *in vivo*, suggesting the expression of VEGFR-2 in podocytes [123]. Podocyte-derived VEGF has a well-documented paracrine function on endothelial cells as well as an autocrine function on podocytes themselves. However, several groups disagree with the idea of podocyte-VEGFR-2 mediating the autocrine function of VEGF. Nevertheless, all these data indicate not only that podocytes possess a functional autocrine VEGF-A signaling through VEGFR-2 but also that VEGF-A signaling regulates the expression of slit diaphragm proteins. Additionally, our studies do not exclude the possible effects of podocyte VEGF_164_ overexpression through VEGFR-1, which is able to regulate in a negative fashion the activity of VEGF and thereby preventing VEGF binding to VEGFR-2. 

 Although the podocyte has been described to be the site of VEGF production within the glomerulus, the role and/or interaction of podocyte-derived VEGF-A in glomerular health and disease still remain debatable. In view of the fact that VEGF receptors (VEGFR-1 and VEGFR-2) are expressed at the highest levels on glomerular endothelial cells, investigations have primarily focused on the paracrine functions for podocyte-derived VEGF-A. At present, several studies demonstrated that VEGFR-2 is expressed at low levels by podocytes and highlight the existence and importance of autocrine podocytic VEGF-VEGFR system in normal and injured podocytes. Disruption or an imbalance of podocyte-derived VEGF-A produces changes in slit diaphragm proteins, and, as consequence, it leads to podocyte effacement and proteinuria. Loss or failure of podocytes function contributes to the development of glomerulosclerosis, which is the final stage of various renal diseases. 

 The biology of the VEGF-VEGFR system is indeed complex. Any manipulation of this system has to be cautious in order not to disrupt its fragile balance. To find a therapeutic strategy is going to be challenging, especially considering the vast diversity of VEGF isoforms and because specific compounds interfering with the VEGF-VEGFR system are limited. Although data is currently unavailable in humans, several strategies to either inhibit or enhance the VEGF axis have shown promising results in animal models of renal disease.
